# Involvement of Reactive Oxygen Species in the Hepatorenal Toxicity of Actinomycin V In Vitro and In Vivo

**DOI:** 10.3390/md18080428

**Published:** 2020-08-15

**Authors:** Fu-juan Jia, Zhuo Han, Jia-hui Ma, Shi-qing Jiang, Xing-ming Zhao, Hang Ruan, Wei-dong Xie, Xia Li

**Affiliations:** 1 Marine College, Shandong University, Weihai 264209, China; jfj1996@outlook.com (F.-j.J.); hanzhuo1013@gmail.com (Z.H.); sdumjh@hotmail.com (J.-h.M.); zoemh48@gmail.com (S.-q.J.); xingming1996@outlook.com (X.-m.Z.); bywh0827@outlook.com (H.R.); wdxie@sdu.edu.cn (W.-d.X.); 2 School of Pharmaceutical Sciences, Shandong University, Jinan 250012, China

**Keywords:** actinomycin D, actinomycin V, hepatorenal toxicity, oxidative stress

## Abstract

The high toxicity of actinomycin D (Act D) severely limits its use as a first-line chemotherapeutic agent in the clinic. Actinomycin V (Act V), an analog of Act D, exhibited strong anticancer activity in our previous studies. Here, we provide evidence that Act V has less hepatorenal toxicity than Act D in vitro and in vivo, associated with the reactive oxygen species (ROS) pathway. Compared to Act D, Act V exhibited considerably stronger sensitivity for cancer cells and less toxicity to human normal liver LO-2 and human embryonic kidney 293T cells using the MTT (3-(4,5-dimethyl-2-thiazolyl)-2,5-diphenyl-2-H-tetrazolium bromide) assay. Notably, Act V caused less damage to both the liver and kidney than Act D in vivo, indicated by organ to body weight ratios, as well as alanine aminotransferase (ALT), aspartate aminotransferase (AST), and serum creatinine (Scr) levels. Further experiments showed that the ROS pathway is involved in Act V-induced hepatorenal toxicity. Act V generates ROS and accumulates malondialdehyde (MDA), reducing levels of superoxide dismutase (SOD) and glutathione (GSH) in LO-2 and 293T cells. These findings indicate that Act V induces less hepatorenal toxicity than Act D in vitro and in vivo and merits further development as a potential therapeutic agent for the treatment of cancer.

## 1. Introduction

Actinomycin D (Act D), extracted and separated from the marine-derived actinomycete *Streptomyces sp*. [1], is a chemotherapy medication that inhibits RNA synthesis [2]. It has been used for the treatment of multiple cancer types, including Wilms tumor [3], rhabdomyosarcoma [4], Ewing’s sarcoma [5], trophoblastic neoplasms [6] and certain types of ovarian cancer [7]. However, clinical treatment with Act D is often limited by its undesirable side effects, such as hepatotoxicity [8] and renal injury [9].

**Figure 1:**
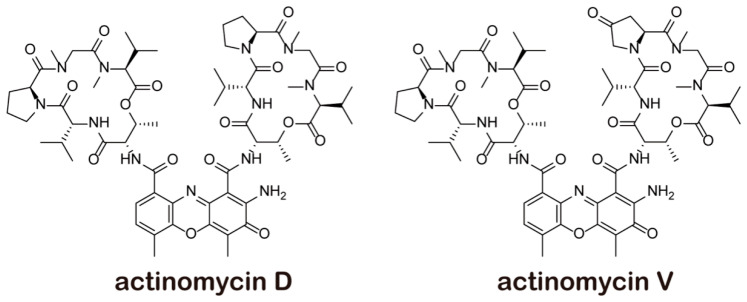
Structure of actinomycins.

Actinomycin V (Act V), which is also extracted from the marine-derived actinomycete *Streptomyces* sp. [1], has a similar structure to that of Act D (Figure 1). In our previous studies [10,11], Act V was found to be more cytotoxic to tumor cells than Act D. Moreover, Act V inhibited the migration and metastasis of breast cancer cells. In this aspect, Act V may be a potential substitute for Act D, so understanding the toxicological mechanisms of Act V and Act D is of great worth.

In an in vitro experiment, it can be difficult to observe drug toxicity to organs in the absence of the body’s overall regulation [12,13]. Mammalian models give integrated results, but the mechanism of toxicity cannot be tested [14]. Therefore, a combination of rodent experiments and cell experiments is the standard method for evaluating drug toxicity [15].

Liver and kidney damage is often accompanied by oxidative stress [16,17], which is an imbalance of oxidative antioxidants caused by the excessive production of reactive oxygen species (ROS) in cells beyond the capacity of cell clearance mechanisms [18]. It is generally characterized by the reduction of antioxidants, such as superoxide dismutase (SOD) and glutathione (GSH), and an increase in oxides, such as malondialdehyde (MDA). In this study, changes in ROS, SOD, GSH, and MDA levels in normal human liver and kidney cells after treatment with Act D and Act V were detected to assess the role of oxidative stress in hepatorenal toxicity.

## 2. Results

### 2.1. Comparing Cytotoxicity of Act V and Act D in LO-2 and 293T Cells

**Figure 2:**
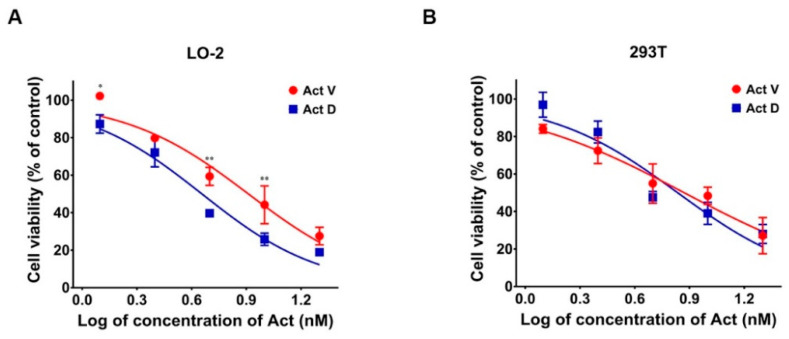
Actinomycin V (Act V) and Act D dose-dependently inhibited the proliferation of LO-2 (**A**) and 293T (**B**) cells. Cells were treated with various concentrations of actinomycins (0 to 0.02 μmol/L) for 48 h. Cell viability was calculated by comparing with the control group (actinomycin 0 nmol/L). The experiment was repeated three times, * *p* < 0.05; ** *p* < 0.01 vs. Act D.

The cells were treated with serial concentrations of Act V and Act D (0–0.02 μM) for 48 h, and the IC_50_s were determined (Act D, which has been used for a long time in clinic, serves as a positive control here), as shown in Table 1 and our previous study [10,11]. Act V exhibited considerably stronger efficacy against cancer cells with a ten-fold lower IC_50_ value compared to Act D. Act V and Act D induced cytotoxicity in human normal liver LO-2 and human embryonic kidney 293T cell lines in a concentration-dependent manner (Figure 2).

### 2.2. Effect of Act V and Act D on Organ to Body Weight Ratios in Mice

**Figure 3:**
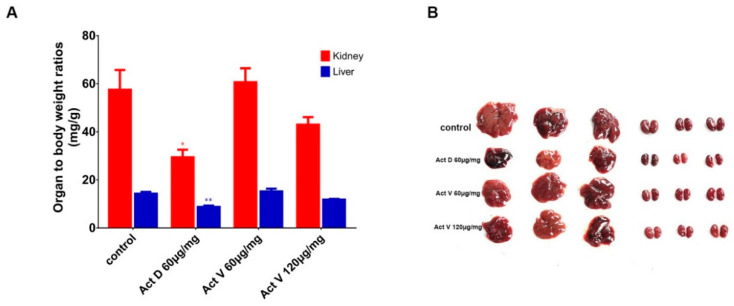
Effect of actinomycins on organ to body weight ratios and liver and kidney size. (**A**) Organ weights were collected and normalized to terminal body weights for the kidney and liver. Significant differences compared to control are indicated as follows: * *p* < 0.05; ** *p* < 0.01. (**B**) The livers and kidneys of mice were taken out after dissection for observation.

The effect of actinomycins on organ to body weight ratios in the four groups of mice is shown in Figure 3A. We set the dosage of Act D to 60 μg/kg, referring to a previous study [19]. All of the mice were injected intraperitoneally for one week. Act V (120 μg/kg) induced a decrease in liver (*p* < 0.05) and kidney (*p* < 0.01) to body weight ratios compared to the control group; however, this effect was weaker than that of Act D (60 μg/kg). Act V (60 μg/kg) induced no significant changes in the organ to body weight ratios. These results indicate that Act D caused more serious damage to the liver and kidney than Act V.

### 2.3. Effect of Act V and Act D on Liver and Kidney Histopathology

**Figure 4:**
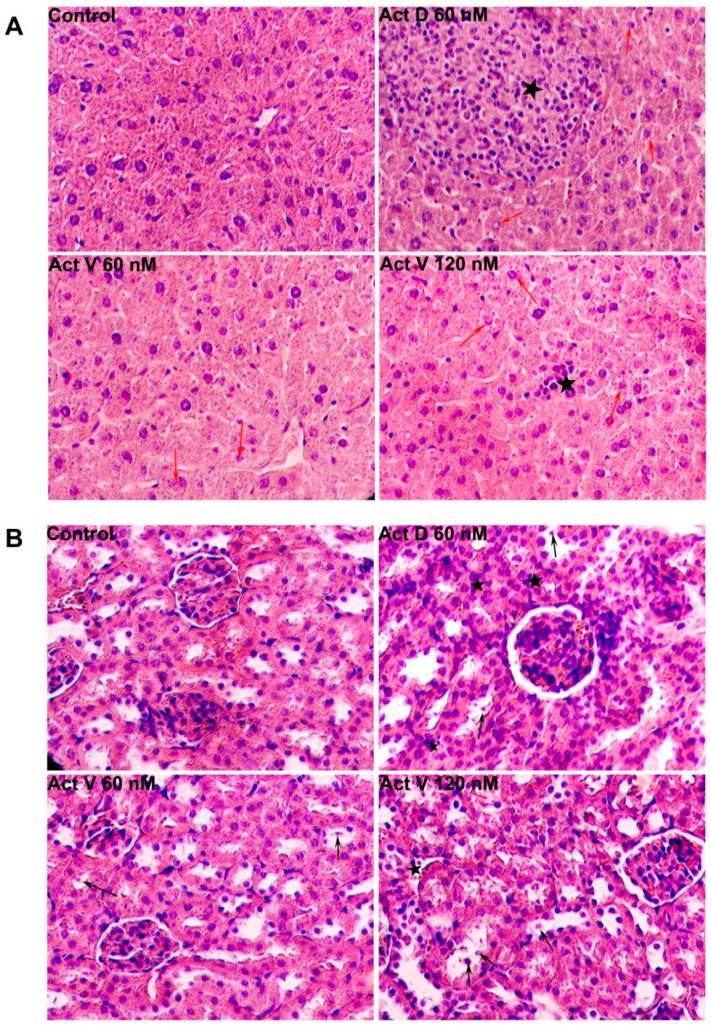
Photomicrographs of (**A**) liver and (**B**) kidney histology (× 200). Asterisks indicate the inflammatory cell infiltration, red arrows indicate hepatocyte edema, black arrows indicate tubular epithelial cell necrosis.

Biochemical analyses are shown in Figure 4. Compared with the control group, significant inflammatory cell infiltration (asterisks) and hepatocyte edema (red arrow) were observed in the liver sections of mice given Act D (60 μg/kg), but little hepatocyte edema and only slight inflammation were observed in the Act V (120 μg/kg) group, and less hepatocyte edema was found with the lower dose of Act V (60 μg/kg). For the kidneys, the Act D (60 μg/kg) group showed marked interstitial inflammatory cell infiltration (asterisk) and tubular epithelial cell necrosis (black arrow), as well as marked Bowman’s capsule expansion. Necrosis of renal tubular epithelial cells was less observed in the Act V (120 μg/kg, 60 μg/kg) groups. These histopathological data further show that Act V induces less liver and kidney damage than Act D.

### 2.4. Effect of Act V and Act D on Liver and Kidney Indicators in Mouse Serum

**Figure 5:**
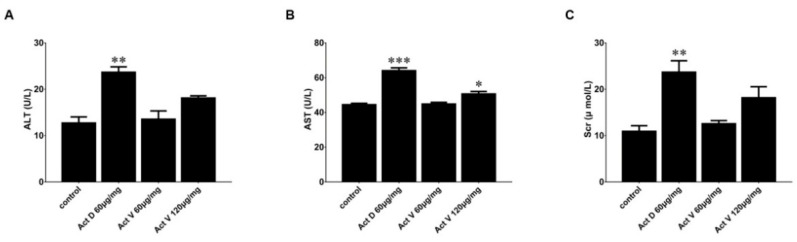
Liver and kidney biochemical indicators in serum after actinomycin administration. Hepatic biochemical indicators: (**A**) alanine aminotransferase (ALT); (**B**) aspartate aminotransferase (AST), renal biochemical indicator; (**C**) serum creatinine. Significant differences compared to control are indicated as follows: * *p* < 0.05; ** *p* < 0.01; *** *p* < 0.001.

Alanine aminotransferase (ALT) and aspartate aminotransferase (AST) are liver enzymes, and serum creatinine (Scr) is an indicator of kidney function. Figure 5 presents the effect of Act V and Act D administration on liver enzymes and creatinine in mouse serum. Compared with the control group, the levels of ALT, AST and Scr were significantly increased in the Act D group, but no such elevations were found in the Act V (60 μg/kg) group. Act V (120 μg/kg) induced a significant increase in AST and a tendency toward increased ALT and Scr, suggesting that the hepatorenal toxicity of Act V at higher doses should be assessed in future drug development.

### 2.5. Act V and Act D Promote the Generation of ROS in LO-2 and 293T Cells

**Figure 6:**
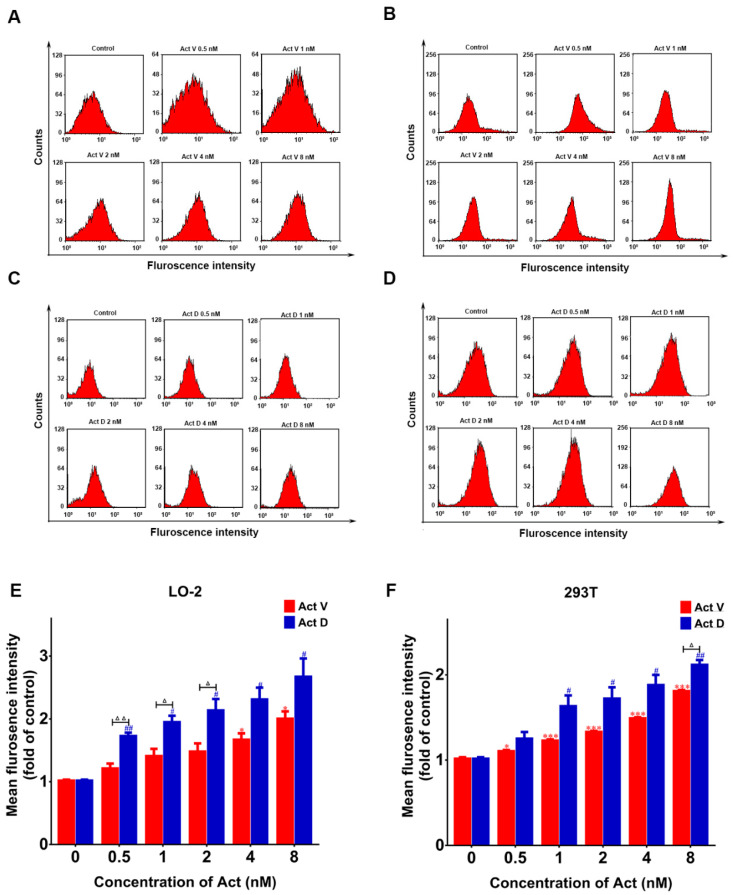
Actinomycin induces increased reactive oxygen species (ROS) levels. Flow cytometry was used to obtain the peak figure of ROS level after actinomycin (0–8 nM) was added to cells and incubated for 24 h: (**A**) Act V in LO-2; (**B**) Act V in 293T; (**C**) Act D in LO-2; (**D**) Act D in 293T; (**E**) ROS increase induced by actinomycin in LO-2 cells compared with the control group; (**F**) ROS increase induced by actinomycin in 293T cells compared with the control group. Experiments were repeated three times: * *p* < 0.05; *** *p* < 0.001 vs. Act V 0 nM; ^#^ *p* < 0.05; ^##^ *p* < 0.01 vs. Act D 0 nM; ^△^ *p* < 0.05; ^△△^ *p* < 0.01 vs. Act D.

The 2′,7′-dichlorodihydrofluorescein diacetate (DCFH-DA) probe can be hydrolyzed by cellular enzymes to form DCFH. This substance can react with ROS to produce fluorescent DCF, so the DCFH-DA probe was used to estimate intracellular ROS production via flow cytometry. As shown in Figure 6, LO-2 and 293T cells treated with Act V and Act D demonstrated a dose-dependent increase in ROS production compared to the control group.

### 2.6. Act V and Act D Accelerate Lipid Oxidation in LO-2 And 293T Cells

**Figure 7:**
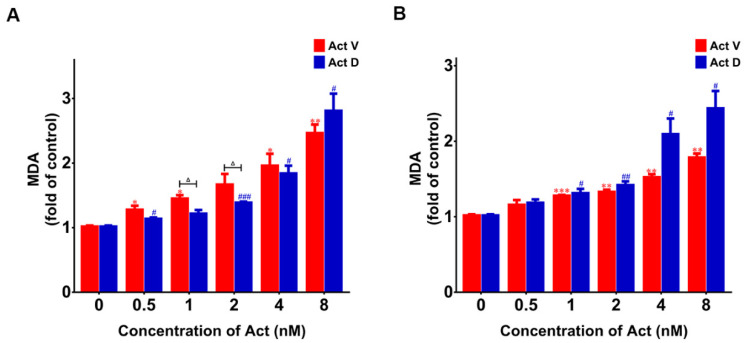
Actinomycin-induced lipid oxidation. Results obtained by assessing malondialdehyde (MDA) levels. (**A**) Actinomycin-induced lipid oxidation in LO-2 cells showed multiple increases compared with the control group. (**B**) Actinomycin-induced lipid oxidation in 293T cells showed multiple increases compared with the control group. Experiments were repeated three times: * *p* < 0.05; ** *p* < 0.01; *** *p* < 0.001 vs. Act V 0 nM; ^#^ *p* < 0.05; ^##^ *p* < 0.01; ^###^ *p* < 0.001 vs. Act D 0 nM; ^△^ *p* < 0.05 vs. Act D.

Lipid oxidation occurs when cells undergo oxidative stress. The level of lipid oxidation can be tested by detecting the level of MDA. The results show that the MDA content in LO-2 and 293T cells treated with Act V and Act D was significantly increased in a concentration-dependent manner compared with the control group (Figure 7). The results suggest that actinomycin may break the oxidative stress balance through upregulating MDA levels.

### 2.7. Act V and Act D Reduce Radical Scavengers Levels in LO-2 and 293T Cells

**Figure 8:**
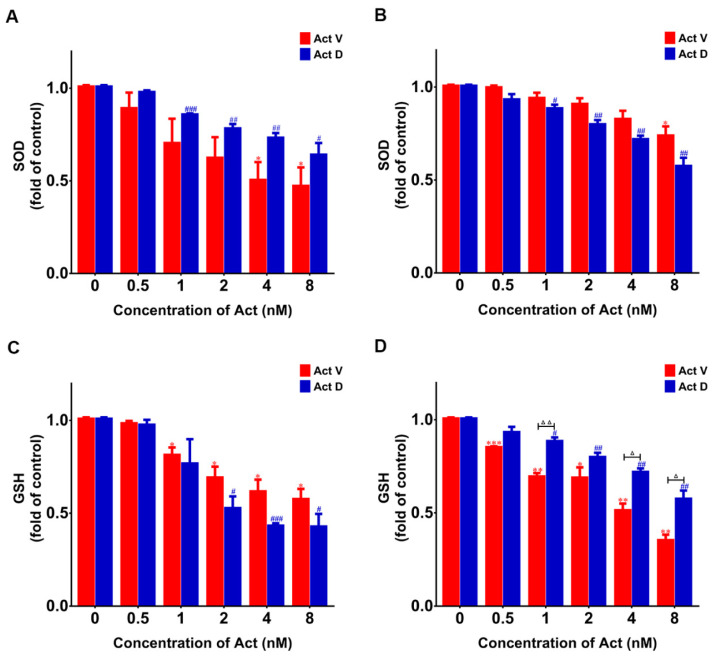
Act D down-regulated superoxide dismutase (SOD) and glutathione (GSH) levels. Results obtained using SOD and GSH kits. (**A**) Compared with the control group, actinomycin reduced SOD in LO-2 cells. (**B**) Compared with the control group, actinomycin reduced SOD in 293T cells. (**C**) Compared with the control group, actinomycin reduced GSH in LO-2 cells. (**D**) Compared with the control group, actinomycin reduced GSH in 293T cells. Experiments were repeated three times: * *p* < 0.05; ** *p* < 0.01; *** *p* < 0.001 vs. Act V 0 nM; ^#^ *p* < 0.05; ^##^ *p* < 0.01; ^###^ *p* < 0.001 vs. Act D 0 nM; ^△^ *p* < 0.05; ^△△^ *p* < 0.01 vs. Act D.

As radical scavengers, SOD and GSH will be reduced in the case of oxidative damage. As shown in Figure 8, at the higher doses of Act D and Act V, the levels of SOD and GSH in LO-2 and 293T cells gradually decreased. These data indicate that increase of ROS production might induce the upregulation of MDA and downregulation of GSH or SOD levels.

## 3. Discussion

Act D has long been used as a clinical antitumor drug, but its hepatorenal toxicity is a serious side effect. Structural optimization and development of homologous actinomycins are two approaches to obtain antitumor agents. This study compared the hepatorenal toxicity of Act D and its homologous compound Act V. The mechanism of hepatorenal toxicity was preliminarily studied, which is conducive to the further study of Act V, a new drug of marine origin.

In vivo experiments can be used to effectively evaluate the efficacy and safety of drugs. The effect of agents on different organs and agent targets can be observed by dissecting mice. Animal experiments can also be used to determine the safe dose range of an agent. Mice were treated with actinomycin for a week, showing that Act D was more toxic than Act V at the same dose, even more toxic than twice the dose of Act V. Act D caused atrophy of liver and kidney tissues, obvious inflammation and cell degeneration and necrosis, and expanded Bowman’s capsule. These results show that mice exposed to Act D display a pronounced impairment in liver and kidney function, which was confirmed by the increased ALT, AST, and creatinine levels in serum. ALT and AST can be assayed in various cells, especially in liver cells. Under normal conditions, serum contains a low content of ALT and AST; however, when liver cells are damaged, membrane permeability increases, and ALT and AST in the cytoplasm is released into the blood, which increases the concentration in serum. The assessment of function of the liver is associated with the determination of ALT and AST activity in serum; i.e., an increase in ALT and AST is regarded as a sign of liver injury. Creatinine is a product of muscle metabolism. Creatinine is excreted mainly through glomerular filtration. The more creatinine in serum, the greater the impairment in glomerular filtration. Act D significantly increased the levels of ALT, AST, and creatinine in serum. In contrast, Act V induced less damage, even at twice the dose of Act D. Therefore, Act V may be an excellent alternative to Act D. The MTT assay confirmed this, showing greater cytotoxicity of Act V in human non-small-lung carcinoma cells (A549), human breast cancer cells (MDA-MB-231), and human leukemia cells (K562), and less cytotoxicity of Act V in human normal liver cells (LO-2) and human embryonic kidney cells (293T).

Oxidative stress refers to the imbalance between oxidative and antioxidant effects in cells and is considered to be one of the most important mechanisms of hepatorenal toxicity [20,21]. When hepatocytes and nephrocytes are stimulated by agents, ROS will accumulate in large quantities and then trigger oxidative stress. The unbalanced oxidoreduction state will further produce more ROS [22]. SOD is the main antioxidant enzyme in the body; it is a scavenger of oxygen free radicals [23]. GSH can clear the peroxide metabolites in cells and block the chain reaction of lipid peroxidation, protecting the structure and function of cells [24]. When the activity of SOD and GSH is reduced, ROS accumulate in liver and kidney cells, and the cell membrane can be easily oxidized by ROS, leading to lipid peroxidation [25]. MDA is a decomposition product of lipid peroxidation and is one indicator of the level of oxidative damage in tissue [26]. When the body suffers from oxidative damage, intracellular ROS and MDA levels will rise, while SOD and GSH levels will decrease. This study indicates that ROS and MDA were obviously increased, and GSH and SOD were obviously decreased after the administration of actinomycin, suggesting that Act V and Act D induce oxidative stress, which could lead to liver and kidney toxicity.

To conclude, the present study confirmed previous research that Act D induces hepatorenal toxicity. Importantly, it also revealed that the mechanism of Act D and Act V toxicity is related to oxidative stress and showed that Act V is only mildly toxic and might be a substitute for Act D.

## 4. Materials and Methods

### 4.1. Reagents

Powdered Act V and Act D (purity 98%, obtained from Dr. Xie at Shandong University) and dimethylsulfoxide (DMSO) were prepared for a 1 μmol/L and 10 μmol/L stock solution, diluted with the cell culture medium in the samples. MTT was acquired from Sigma-Aldrich Corp. (St. Louis, MO, USA). Reactive Oxygen Species Assay Kit (S0033S), Lipid Peroxidation MDA Assay Kit (S0131S), Total Glutathione Peroxidase Assay Kit (S0058), Total Superoxide Dismutase Assay Kit with NBT (nitroblue tetrazolium) (S0109), and Hematoxylin and Eosin Staining Kit (C0105) were obtained from the Beyotime Institute of Biotechnology (Shanghai, China). Alanine aminotransferase Assay Kit (C009-2-1), Aspartate aminotransferase Assay Kit (C010-2-1), and Creatinine (Cr) Assay Kit (sarcosine oxidase) (C011-2-1) were obtained from Nanjing Jiancheng Bioengineering Institute (Nanjing, Jiangsu, China).

### 4.2. Animals

Twelve six-to eight-week-old male Kunming mice and their feed were purchased from Qingdao da-ren-Fucheng Co., LTD., mice were adapted to the environment under standard laboratory conditions for two weeks before the experiment began. All animal studies were approved by the Laboratory Animal Ethical and Welfare Committee of Shandong University Cheeloo College of Medicine (permit no. 18013), and all procedures were conducted in accordance with the guidelines of Shandong University Cheeloo College of Medicine.

### 4.3. Treatments and Sample Collection

Twelve mice were randomly divided into four groups (*n* = 3): the control group (100 mL saline); group 2 (Act D 60 μg/kg, dissolved in 100 μL saline); group 3 (Act V 60 μg/kg, dissolved in 100 μL saline); group 4 (Act V 120 μg/kg, dissolved in 100 μL saline). All the mice were given an intraperitoneal injection daily for one week. Body weight was determined before culling, and the liver and kidney were weighed after dissection. The relative organ weight (%) was calculated according to previous reports [27]. Part of the liver and one of the kidneys were placed in 10% buffered formalin. Blood was collected from the retro-orbital plexus and transferred into centrifuge tubes loaded with heparin sodium, then left to stand for 30 min and centrifuged at 3000 rpm for 20 min. The serum was removed and stored at 4 °C.

### 4.4. Histopathological Examination

The liver and kidney were embedded in paraffin, sectioned, stained by the Hematoxylin and Eosin Staining Kit, as instructed, and observed under the microscope.

### 4.5. Estimation of Liver and Kidney Function Markers in Mouse Serum

An Alanine aminotransferase Assay Kit and Aspartate aminotransferase Assay Kit were used to evaluate liver damage, and a Creatinine (Cr) Assay Kit (sarcosine oxidase) was used to evaluate kidney damage. All the kits were used following standard methods.

### 4.6. Cell Lines

The human normal liver cell line LO-2 and human embryonic kidney cell line 293T were obtained from the Shanghai Institute for Biological Sciences (SIBS), Chinese Academy of Sciences (Shanghai, China) and were cultured according to the supplier’s instructions.

### 4.7. MTT Assay

The inhibition of LO-2 and 293T cell proliferation by Act V and Act D was detected using the MTT assay. LO-2 and 293T cells were seeded in 96-well plates (5 × 10^3^ cells per well). Following cell adhesion, we added 2.4 μL drug (10 μmol/L) into 1.2 mL medium (5% FBS (fetal bovine serum) without DMSO (dimethyl sulfoxide)) as a 0.02 μmol/L drug initial solution (finally containing 0.2% DMSO). The medium (5% FBS and 0.2% DMSO) was then used to dilute the other doses’ compounds and was also used in the control group. After 48 h incubation, 15 μL of MTT (5 g/L) was added in each well to react to form formazan over a period of 4 h. Then, the medium was removed, and 150 μL of DMSO was added per well. The absorbance of the wells was measured using a Microplate Reader at 570 nm. The IC_50_ values of each cell line were calculated by comparing untreated cells, which were presumed to represent 100% cell survival. Each test was conducted in triplicate independently.

### 4.8. Flow Cytometric Analysis of ROS Levels

To measure the actinomycin-induced ROS level change in LO-2 and 293T cells, we performed flow cytometry using a Reactive Oxygen Species Assay Kit. Cells were seeded in 6-well plates at a density of 5 × 10^4^ cells per well. Following adhesion, we added 8 μL Act V (1 μmol/L) and Act D (1 μmol/L) into 1 mL medium (5% FBS without DMSO) as a 8 nmol/L drug initial solution (finally containing 0.8% DMSO). The medium (5% FBS and 0.8% DMSO) was then used to dilute the other doses’ compounds and was also used in the control group. After 24 h incubation, cells were collected and washed with PBS; then, the Reactive Oxygen Species Assay Kit was used according to the manufacturer’s instructions.

### 4.9. Measurement of MDA, GSH and SOD Content

A Lipid Peroxidation MDA Assay Kit, Total Glutathione Assay Kit, and Total Superoxide Dismutase Assay Kit with NBT were used to detect the content of MDA, GSH, and SOD in LO-2 and 293T cells after the administration of Act V and Act D in various concentrations. Cells were seeded in 6-well plates at a density of 5 × 10^4^ cells per well. Following adhesion, we added 8 μL Act V (1 μmol/L) and Act D (1 μmol/L) into 1 mL medium (5% FBS without DMSO) as a 8 nmol/L drug initial solution (finally containing 0.8% DMSO). The medium (5% FBS and 0.8% DMSO) was then used to dilute the other doses’ compounds and was also used in the control group. After 24 h incubation, cells were collected and washed with PBS, then, the kits were used according to the manufacturer’s instructions.

### 4.10. Statistical Analysis

Data are presented as mean ± standard deviation from triplicate experiments. All data were analyzed using one-way analysis of variance (ANOVA) for multiple comparisons by SPSS 16.0 (SPSS Inc., Chicago, IL, USA). Significant differences are indicated as follows: * *p* < 0.05; ** *p* < 0.01; *** *p* < 0.001.

## Figures and Tables

**Table 1:** Cytotoxicity of actinomycins in different cell lines.

Cell Lines IC_50_ (μmol/L)	Act V	Act D
LO-2	0.0828 ± 0.0015	0.0382 ± 0.0015
293T	0.0750 ± 0.0008	0.0675 ± 0.0025
A549	0.0068 ± 0.0006	0.1284 ± 0.0072
MDA-MB-231	0.0083 ± 0.0032	0.1515 ± 0.0052
K562	0.0046 ± 0.0001	0.1272 ± 0.0033

Cytotoxicity of actinomycins in various cell lines. Cells were treated with various concentrations of actinomycin V (Act V) and actinomycin D (Act D) and measured via the MTT (3-(4,5-dimethyl-2-thiazolyl)-2,5-diphenyl-2-H-tetrazolium bromide) assay after 48 h. Data are presented as the mean ± SD of three independent experiments.
